# Government policies on job satisfaction of radiographers in tertiary hospitals, Gauteng

**DOI:** 10.4102/hsag.v29i0.2502

**Published:** 2024-06-12

**Authors:** Thandokuhle E. Khoza

**Affiliations:** 1 Department of Radiography, Faculty of Health Sciences, Durban University of Technology, Durban, South Africa

**Keywords:** radiographers, job satisfaction, government policies, occupational-specific dispensation, *Employment Equity Act*, performance management, development system

## Abstract

**Background:**

The National Department of Health has different operational policies to monitor the performance of its employees and to reward them accordingly. These policies could have a direct bearing on job satisfaction and career advancement for radiographers employed by the public sector, as there are previous reports that show that these policies affect the job satisfaction of other healthcare professionals negatively.

**Aim:**

The aim of the study was to explore the influence of government policies on the job satisfaction of radiographers employed by public tertiary hospitals in the Gauteng province.

**Setting:**

The study was conducted in two public tertiary hospitals in the Gauteng province.

**Methods:**

The study used a primary exploratory qualitative research design, with a purposive sampling technique. Data were collected through individual and focus group interviews. The researcher recorded and transcribed the interviews. Thematic analysis was used to analyse the data.

**Results:**

Three government policies had a negative impact on job satisfaction for radiographers. These were the Occupational Specific Dispensation (OSD), the Performance Management and Development System (PMDS) and the *Employment Equity Act (EEA)*. The failure to effectively implement these policies also had a negative bearing on career pathing.

**Conclusion:**

The implementation and structure of these three government policies have resulted in job dissatisfaction amongst radiographers employed by public tertiary hospitals and reduced the structure of career pathing.

**Contribution:**

The study highlighted three government policies that negatively affect job satisfaction in Gauteng.

## Introduction

Job satisfaction is a positive emotional state resulting from recognition of an individual’s job (Suarez, Asenjo & Sanchez 2017:31). It is also considered to be an aggregate of feelings felt by an individual about their job and the attitude arising when those feelings are well-balanced (Kim, Kim & Kim 2011). Different researchers have identified multiple factors that are known to either negatively or positively affect job satisfaction and these are: supervisor effectiveness (Coomber et al. 2006); staff shortages (Verrier & Harvey 2010; Weng et al. 2010); interpersonal relations at work (Nassar et al. 2011); heavy workload (Coetzee et al. 2013); working hours (Jones, Horwitz & Wilkin 2013); pay (Netshiswinzhe et al. 2015); safe environment (Khamisa et al. 2017); and burnout (Suarez et al. 2017).

It is for this reason that Herzberg’s motivation and hygiene theory categorises the elements of job satisfaction into intrinsic and extrinsic factors. The intrinsic factors are motivating and result in job satisfaction. The extrinsic factors are hygienic and may result in job dissatisfaction if not implemented (McGrath & Bates 2013). Intrinsic factors include recognition, achievement, advancement, nature of the work undertaken and responsibility. Extrinsic factors include pay, company policies, relationship with supervisors, working conditions and feelings associated with a lack of status or security (McGrath & Bates 2013). The success of implementing hygiene factors is dependent on the following five rules: Identify the type of hygiene factor; Provide purpose for the hygiene factor; Keep the administration of hygiene factors simple; Give the hygiene factor and do not show off about it; and lastly give the hygiene factors according to performance.

The Gauteng Department of Health uses the Performance Management Development System (PMDS) to monitor and reward hygiene factors, which was introduced in 2001. This policy aimed to plan, manage and improve employee performance through financial recognition linked to their PMDS (DPSA 2007). The PMDS ratings are decided by line managers assigned to employees in each financial year. However, previous studies have shown that line managers lack the required skills and expertise to effectively rate their subordinates (Mashego & Skaal 2016). Therefore, this could hinder the recognition of performance and its associated financial rewards. As a result, the PMDS has led to employee demotivation, and the authors are of the view that the tool is outdated and failed to provide employees with a platform for skill development (Adejoka & Bayat 2014).

In addition to extrinsic factors of job satisfaction that are linked to company policies, public sector hospitals have the Occupational Specific Dispensation (OSD) policy. The National Department of Health (DOH) in 2010 introduced the OSD policy for therapeutic, diagnostic and allied health professionals. The objectives of the OSD were to introduce career pathing opportunities based on competencies, experience and performance; provide grade progressions within the limits of the relevant grades based on performance; recognition of appropriate experience for the purpose of grade progression; the recognition of performance for accelerated progression to higher grades and pay progression within a salary grade; and differentiated salary scales for identified categories of professionals based on a new remuneration structure (PHSDSBC 2010).

The implementation of this policy resulted in increased numbers of nurses employed by the public sector (Coetzee et al. 2010), and nurses with higher salary packages expressed higher levels of job satisfaction (Netshiwinzhe & Mulaudzi 2015). However, the introduction of OSD might have improved the salaries of healthcare professionals employed by public hospitals, but they are still not compatible with international salary scales (George et al. 2012:3). Therefore, this study aimed to explore the influence of government policies on job satisfaction and career pathing amongst radiographers employed by public tertiary hospitals in the Gauteng province, South Africa. The study was guided by Herzberg’s motivation-hygiene theory to explore if any of the policies that govern radiographers employed by public hospitals may have a negative bearing on job satisfaction.

## Research methods and design

### Research design

This study used a primary exploratory qualitative research design to explore the phenomenon of job satisfaction amongst radiographers employed in two tertiary hospitals in Gauteng province. In this study, two methods of data collection were used: individual interviews and focus group interviews.

### Study setting

The study was conducted in two public tertiary hospitals in the Gauteng province between November 2018 and June 2019. The two tertiary hospitals were chosen as they employ all five disciplines of radiography, which are diagnostic radiography, radiation therapy, sonography, nuclear medicine radiography and mammography.

### Ethical considerations

Ethical clearance to conduct the study was obtained from the Durban University of Technology, IREC 115/18. The gatekeeper’s permission was obtained from the Gauteng Department of Health GP201808_044. At the hospital level, the study was approved by the heads of Radiology and a permission letter was signed off by the Chief Operating Officer. In one of the hospitals, a teaching hospital at the University of Pretoria special ethical clearance, Ethic Reference 643/2018, had to be obtained from the University as it is associated with this institution. Participants had to sign a consent form to permit the audio recording of the interviews by the researcher.

### Study population and sampling

Purposive sampling was used to identify participants who were employed by the two tertiary hospitals at the time of data collection. For the purposes of this study, the term radiographer is used to describe the five disciplines within radiography. Therefore, the sample had to include all the different disciplines within radiography and the different levels of experience as a qualified radiographer.

### Data collection

Once all the relevant gatekeepers’ permissions were obtained, the researcher was invited to departmental meetings to introduce the aim of the research and its aims. This platform was also used as a method to recruit participants. Interested participants were asked to contact the researcher via email. Once the number of participants met the requirements of focus group interviews, arrangements were made with participants in terms of time, date and venue to conduct the interviews. The study purposefully selected one participant per discipline to ensure that opinions were received from all the disciplines of radiography.

Data collection occurred in two forms, focus group interviews and individual interviews. One focus group interview was conducted in each hospital, and all the disciplines were invited to partake. The focus group interviews comprised radiographers from the five disciplines with different levels of experience, ranging from grade 1 to 3; different genders; and different age groups. In one of the hospitals, there were five participants, whereas the other one had four participants as demonstrated in Table 1 and Table 2. The focus group interviews were scheduled for the afternoon, to minimise interruptions in the workflow. On average, the focus group interviews took one and a half hours to conduct. They were conducted in the department’s boardroom, and they were audio-recorded by the researcher with the permission of the participants. In each of the interviews, the researcher used a standard interview guide and asked probing questions based on the responses. The interview sessions were closely monitored to ensure that all the participants were active during the interview sessions.

**TABLE 1:** Focus group interview 1.

Participants	Gender	Age (years)	Experience (years)	Position	Discipline
1	Male	21–33	1–10	Grade 1	Diagnostic
2	Male	34–49	1–10	Grade 1	Nuclear medicine
3	Female	21–33	1–10	Grade 1	Sonographer
4	Female	34–49	1–10	Grade 1	Radiation therapy
5	Female	> 50	> 20	Grade 3	Mammography

*Source*: Khoza, T.E., 2020, ‘A model to enhance job satisfaction for radiographers employed at the selected public tertiary hospitals in the Gauteng province, South Africa’, PhD thesis, Dept. of Health Sciences, Durban University of Technology

**TABLE 2:** Focus group interview 2.

Participants	Gender	Age (years)	Experience (years)	Position	Discipline
6	Female	21–33	1–10	Grade 1	Mammography
7	Female	21–33	1–10	Grade 1	Diagnostic
8	Female	21–33	1–10	Grade 1	Radiation therapy
9	Female	34–49	10 ≥ 20	Grade 2	Nuclear medicine

*Source*: Khoza, T.E., 2020, ‘A model to enhance job satisfaction for radiographers employed at the selected public tertiary hospitals in the Gauteng province, South Africa’, PhD thesis, Dept. of Health Sciences, Durban University of Technology

Individual interviews were used for heads of departments because of their positions; this means they have busy schedules and would have made it challenging to coordinate focus group interviews. The timings of the interviews were scheduled according to the availability of the managers, and they were conducted in the offices of the managers. The length of the interviews ranged from 45 min to 1 h. All the different disciplines were represented as shown in Table 3. It is worth noting that some of the disciplines had one-liner managers; they all reported to the Diagnostic Radiography assistant director. These disciplines were mammography, diagnostic radiography and sonography. With the permission of the managers, the interviews were recorded by the researcher.

**TABLE 3:** Individual interviews.

Participants	Gender	Age (years)	Experience (years)	Position	Discipline
10	Female	34–49	> 20	Assistant director	Nuclear medicine
11	Male	34–49	> 20	Assistant director	Radiation therapy
12	Female	34–49	10 ≥ 20	Assistant director	Radiation therapy
13	Female	> 50	> 20	Assistant director	Diagnostic
14	Female	> 50	> 20	Assistant director	Diagnostic
15	Male	> 50	> 20	Assistant director	Nuclear medicine
16	Female	> 50	> 20	Assistant director	Sonographer

*Source*: Khoza, T.E., 2020, ‘A model to enhance job satisfaction for radiographers employed at the selected public tertiary hospitals in the Gauteng province, South Africa’, PhD thesis, Dept. of Health Sciences, Durban University of Technology

### Data analysis

Thematic analysis of data was used to identify themes from recorded and transcribed interviews. According to Creswell and Plano Clark (2011), there are five steps to be followed in thematic data analysis, and this is how they were achieved in this study: The first step of data analysis was initiated by organising documents, audio data and transcribing the data, which was done by the researcher. The next process was to explore the data, which was done by reading through the transcribed data and field notes, writing memos and developing a qualitative codebook. Coding was done to ensure the researcher found meaning in each item of data and to construct a pattern in the data (Saldana 2016). The actual analysis of the data was done by coding the data, assigning labels to codes, grouping the codes into themes, and interrelating themes. The final step was presenting the findings in discussions of themes and presenting visual models, figures and tables. This process was continuously repeated to ensure that all the possible codes were entered into a codebook. Once the themes were finalised, the raw data and codebook were submitted to the supervisors of the research project for verification. In addition to this, a quantitative study was conducted to add triangulation, but quantitative results will not be presented in this article. Furthermore, the findings including the themes and codes were shared with participants to confirm if they reflected what was discussed in the group interviews. No analytical software was used to analyse the data.

### Trustworthiness

As qualitative research has an element of subjectivity and is open to criticism, the study and the findings must provide evidence of validity and reliability (Polit et al. 2012:174). Lincoln et al. (1985) suggest that there is an alternative to validity and reliability that would provide the evidence for a decision trail and trustworthiness to be assured within qualitative research. Trustworthiness refers to the extent to which a research study is worth paying attention to, worth taking note of and the extent to which others are convinced that the findings are to be trusted (Babbie et al. 2001). Terms that are associated with qualitative research are trustworthiness, authenticity, dependability and confirmability.

#### Confirmability

Confirmability requires the researcher to minimise bias during data collection to ensure that the findings are supported by data (Brink 2007:118). The authors further add that bias could occur at any part of the research process, which could be intentional or unintentional. Triangulation is a method that is used to conclude the research phenomenon through the use of multiple sources. In this study, the researcher used data triangulation to obtain data from multiple sources and through different methods of data collection. These multiple sources were the representatives from the five disciplines of radiography and their line managers. Data triangulation was achieved through the collection of data using focus group interviews and one-on-one interviews. Once all the themes and sub-themes were generated, an audit trail was conducted to confirm findings, interpretations and recommendations supported by actual data. The themes and sub-themes were presented to some of the participants to verify that they contained what was discussed in the interviews. All these were done to minimise bias from the researcher and the participants. The researcher also employed reflexivity which involves self-awareness and critical self-reflection on the researcher’s potential biases as these may affect the research process and conclusions.

#### Dependability

Dependability requires the researcher to ensure that the findings of the study are consistent and can be repeated (Brink 2007). To ensure dependability, an audit trail was maintained through safe keeping of raw data of each interview for future reference. The audit involves a scrutiny of the data collected and any supporting documentation by the supervisors. Although the researcher coded the data himself, the data and analysis were checked for discrepancies and scrutinised by the research supervisors.

## Results

### Demographics

A total of 16 participants took part in focus group discussions and individual interviews. In one focus group interview, there were five participants from all the disciplines of radiography, and only four participants were in group two as there were no sonographers employed by the hospital at the time of data collection, as demonstrated in Table 1 and Table 2. The sonographer employed in that hospital had recently resigned. A total of seven heads of departments were interviewed, representing each radiography discipline. It is worth noting that mammography radiographers were managed by diagnostic radiography managers, as demonstrated in Table 3. Of the 16 participants, 4 were males and 12 were females.

During thematic analysis, two themes emerged, which were: the influence of government policies on job satisfaction and the lack of career pathing. The guiding research question for this research project was: How do the policies introduced by the Department of Health contribute to job satisfaction for radiographers employed by tertiary hospitals in the Gauteng province?

### Theme 1: The influence of government policies

Three policies were dominant in the focus group discussions and individual interviews, and these were the OSD, PMDS and the *Employment Equity Act (EEA)*.

#### Sub-theme 1.1: Occupational specific dispensation

Different sentiments were shared by participants about the introduction of OSD, and some felt it was unfairly implemented while others thought that it resulted in improved salaries for radiographers. It is worth noting that the policy was introduced on 01 July 2010 for radiographers (PHSDSBC 2010). Its objectives were to introduce career paths based on competencies, experience and performance. Initially, other authors have agreed that the introduction of the OSD policy saw increased salaries for healthcare professionals employed by the public sector, which ultimately increased the number of healthcare professionals employed by the sector (Coetzee et al. 2013; Netshiwinzhe & Mulaudzi 2015). These are extracts that were taken from the interviews:

‘After the OSD I got less money than before because we had like scare skill allowance that was not taxed so when they took that away, they added it to my salary, and it pushed me to a higher tax bracket. The ADs were the only people who had that experience; all the other people at least got more money and the juniors a lot more. But unfortunately for me, the OSD is fooling people because now the entry-level salary is high, but you are going to stay on that salary almost for ten years before you go to the next grade.’ (Participants 14, Female, >50 years)

‘I can assure you the salary is better than private since the OSD. Those who still go out to private and say things are better; are not telling the truth. It is difficult for private to match what OSD is giving.’ (Participant 2, Male, 34 years–49 years)

‘This is something which is a bone of contention because different HR apply it in different ways, so from this department, I must say that when all the requirements were put to me as a manager to complete. I consulted the people like the Chiefs at that time and agreed that this is where you belong and in accordance with that document. Hence we have Chiefs that are on a supervisory level. I don’t know how they made it because other disciplines of radiography are having this very dissatisfaction about it too. However, like even in some of the levels like with the assistant directors, you could see that some salary levels of the AD are lower and others are higher so you never get to really understand it properly.’ (Participant 15, Male, >50 years)

#### Sub-theme 1.2: Performance management development system

The PMDS is a tool used to monitor the performance and development of healthcare professionals employed by the public health sector. The PMDS is also used as an appraisal tool, where supervisors measure radiographers’ performances against set targets (DPSA 2007). It consists of a rating of 1–5, where 1 is not effective and 5 is very effective. However, participants felt that they were not fairly rated, and some of the participants were in agreement with this statement. Previous authors have cited that supervisors lacked the required skills to fairly rate their subordinates (Mashego & Skaal 2016). In addition, other authors have mentioned that the tool was outdated and lacked the platform for skill development (Adejoka & Bayat 2014):

‘I can’t be battling for a 3, because from what I understand, a 3 is me coming to work and completing my duties. I believe that I have one of those jobs where I just have to throw my entire body into it. However, I must go to my manager’s office and now cry, beg and grovel for a 3, it’s a bit of an insult. We hardly get a 5, and someone must die for you to get 5.’ (Participant 7, Female, 21 years–33 years)

‘Well, with the PMDS I do everyone’s PMDS, so I evaluate everyone. There used to be a time when we used to assign junior radiographers to a chief radiographer to evaluate them. But that became a problem, because if the chief radiographer had a problem with a junior radiographer, then they were not fairly evaluated. Then we decided, it’s better if one person does everyone.’ (Participant 12, Female, 34 years–49 years)

‘They don’t like the PMDS, they say it is not fair, which is true and it is not fair to everyone, for example, if I do my own PMDS I’m not going to score myself a three. Because I feel like I’m hard working, I’m going to put a four and put my motivation and get my bonus that’s all I want you to understand. But for others, if you are going to get evaluated by somebody and you give that person a three which is standard, they don’t feel like you’ve evaluated them fairly, they also want to get the fours and fives.’ (Participant 11, Male, 34 years–49 years)

#### Sub-theme 1.3: Employment Equity Act

The *EEA No. 55 of 1998*, was introduced by the South African government to address the discriminatory laws and practices in employment and income within the national labour market (Department of Labour 2013). Statistics indicate that only 20% of Africans are retained in skilled or intermediate occupational levels (Jones et al. 2012). The reported barriers to the implementation of the EEA with regards to retention include a lack of cultural sensitivity where recruits are expected to assimilate into the current organisational culture; a lack of cultural awareness programmes and organisational culture that values diversity; black African people not fully integrated into the organisation with a meaningful delegation of duties; and African staff members not being systematically developed and trained (Booysen 2007). Managers acknowledged the failure to retain previously discriminated groups and also cited additional challenges with retaining them:

‘Because it’s always that the better candidate was missed, and we end up with someone who either doesn’t stay long afterwards or they are troublemakers or they are just lazy and they don’t want to do the work that you want them to do. They don’t like to listen to instructions, and you know the new generation they don’t believe in rules.’ (Participant 11, Male, 34 years–49 years)

‘It is always unfair, for example, we had a post for a chief mammographer, but the person with the experience did not get it because they are not of the right race. Now, they have left and gone to private and what can we do, policy is policy, they won’t even shortlist if the demographics are not right.’ (Participant 13, Female, >50 years)

### Theme 2: Career pathing

There were four sub-themes for career pathing, namely: diversity, academic growth, CPD activities and promotion.

#### Sub-theme 2.1: Diversity of tasks

The different disciplines of radiography consist of sub-sections within them, and rotation within these sub-sections was a significant contributor to job satisfaction. These sub-categories were considered a specialty; they were predominantly for diagnostic radiographers and radiation therapists. In diagnostic radiographers, these are Computed Tomography; Magnetic Resonance Imaging (MRI); Angiography, and Catheterization Laboratory. For radiation therapists, this included working in the treatment planning department. The placement of radiographers in these specialised areas could be a career ladder programme. Career ladder programmes allow employees to gain new skills and knowledge while on the job, and organisations reward them with higher salaries and promotions (Dill, Chuang & Morgan 2014). However, participants cited that there was no additional remuneration for radiographers working in specialised departments:

‘Staff in my department have indicated that if they get CT training, then they would stay. I don’t know what it is about CT, but they really want to be trained in CT. I think for them, it’s like it a modality that which they can feel they can work, it is complex, and they can work independently.’ (Participant 14, Female, >50 years)

‘Honestly salary matters, currently I am CT trained and will be going for catheterization laboratory and MRI training, but salary stays the same. But if you go to private, they will pay a different salary if you are competent in these departments.’ (Participant 1, Male, 21 years–33 years)

‘In varsity, they teach us all things, but we cannot apply them in the clinical setting. The last time I was placed in treatment planning was when I was a student. So, what is the point of me learning all these skills.’ (Participant 4, Female, 34 years–49 years)

‘We make them aware that look we have a list to train in specialized areas like CT most people like CT, because it’s also in attraction for when people want to leave and whenever they go, they would ask them CT. We also tried to say look we want people to work on night duty because when you’re on night duty, we really need people that are trained in CT.’ (Participant 13, Female, >50 years)

#### Sub-theme 2.2: Lack of promotion

The two government policies on PMDS and OSD are directly linked to promotion. According to the OSD policy, radiographers who obtain an above-average performance on their PMDS ratings qualify for accelerated grade progression (PHSDSBC 2010). Accelerated grade progression refers to a move from a lower grade (salary scale) to the next higher grade (salary scale) attached to an OSD post, based on the specific requirements for grade progression and accelerated grade progression in the OSD post (PHSDSBC 2010). Accelerated grade progression will only be applicable after 5 years if a radiographer has been consistently rated as outstanding on four consecutive PMDS assessments. Failure to achieve outstanding performance would result in radiographers being in the same grade for 8 years, where automatic grade progression is applied. However, participants indicated that their accelerated grade progression had not been implemented, and challenges in the financial fiscal were cited as the contributing factor. Participants also felt that the prospects of being promoted were non-existent as there was only one managerial position. Similarly, newly qualified radiographers were excited about their new roles but believed the prospects of being promoted were limited:

‘That’s just the thing with OSD, what they did was they changed the way that you would move up. They tell you if you do your PMDS, you can get accelerated grade progression in 5 years, or else you end up in one grade for ten years. But now, because there is no money, the government has suspended accelerated grade progression until further notice.’ (Participant 14, Female, >50 years)

‘There is one manager, and that’s it. So, someone must die for you to get that post, there’s not that growth, there’s a com serve, and then there’s a second step of being qualified. There’s no growth.’ (Participant 4, Female, 34 years–49 years)

‘Well, in my case, I’m relatively new, so I feel like there is still a lot to learn, but the ceiling looks really close.’ (Participant 8, Female, 21 years–33 years)

#### Sub-theme 2.3: Lack of professional growth

Organisations could enhance employee commitment by helping employees to reach their career goals, acquire new skills and offer financial incentives for their efforts (Weng et al. 2010). Consequently, this could enhance employee retention in an organisation (Ellenbecker & Cushman 2011). However, according to the participants, there were no financial incentives for postgraduate qualifications, which resulted in their demotivation to pursue postgraduate studies. Similarly, radiographers working in specialities within radiography such as mammography and radiation therapy cited that they were not placed in their correct posts according to their qualifications nor their registration with the Health Professions Council of South Africa. In addition, for radiographers in speciality, there were no supervisory posts for them except for the head of the department. This goes against what has been done internationally; in America, new roles for radiographers such as radiologist assistants were introduced in the early 2000s (Williams et al. 2004). It is worth noting that role extension has not been fully integrated into radiographers in South Africa:

‘There is no recognition of postgraduate skills or qualification. I can obtain my Masters in the field, and have a PhD, but I am still like any other radiographer, who’s got a 3-year diploma or sometimes a person with the 3-year diploma is more recognized than I am.’ (Participant 15, Male, >50 years)

‘Because working for the government you don’t get recognized as a mammography radiographer, you are still a diagnostic radiographer with a mammography qualification. So, my pay-slip still says diagnostic radiographer because they don’t have mammography posts.’ (Participant 6, Female, 21 years–33 years)

‘It doesn’t reflect on their pay slips, and it doesn’t align with their registration with HPCSA, because with council it recognizes them as radiation therapist radiographers, but in government hospitals, they are diagnostic radiographers.’ (Participant 15, Male, >50 years)

‘After 3 years, a diagnostic radiographer can apply for a chief post and be absorbed into a chief post. So you find that now those who are there in speciality, once they are there, they start recognizing that it is actually a disadvantage in terms of the remuneration. Look at the OSD notches, those notches for diagnostic radiography have grades 1, 2, and 3. Nuclear medicine as a speciality production-grade 1, 2, and 3.’ (Participant 2, Male, 34 years–49 years)

#### Sub-theme 2.4: Continuous professional development

Continuous professional development comprises learning activities through which healthcare professionals maintain and develop their knowledge and skills throughout their careers to ensure that they retain their capacity to practice safely, effectively and legally within their evolving scope of practice (Elshami et al. 2016). However, participants from a speciality felt the topics were not inclusive and were dominantly created for diagnostic radiographers, thus not worth their time:

‘In the CPD meeting is like 12, 4, 8 hours on a Saturday, which always seems to be at the beginning of the month. But they are not always relevant topics. I know it’s going to sound very bad; if you take, for instance most of the CPD classes you go to, they have one ultrasound topic. Do you want to think I want to waste my whole weekend for one ultrasound topic?.’ (Participant 3, Female, 21 years–33 years)

The CPD activities are further categorised into voluntary, obligatory and mandatory. Mandatory CPD activities are monitored and are linked to penalties if they are not performed (Elshami et al. 2016). In South Africa, as part of mandatory CPD activities, the HPCSA requires radiographers to obtain a minimum of 30 continuing education units in a year, and these must include ethics, human rights and medical law (Naidoo & Naidoo 2018). The timing of the CPD activities was cited as a challenge for some of the participants, resulting in the collapse of structures that organised them. Also, the option to do online CPD activities was becoming a preferred method to obtain CPD activities:

‘Society of Radiographers in Pretoria, it’s like non-existent anymore because people just don’t want to attend things in the evenings. They rather do CPD via the computer, some of these websites you can get CPD points because that was the motivation about the society for us to get together and get interesting speakers to better ourselves and get CPD.’ (Participant 7, Female, 21 years–33 years)

## Discussion

The findings showed that two government policies and the lack of career pathing within the radiography profession negatively affected job satisfaction among radiographers employed by public tertiary hospitals within the Gauteng province in South Africa. One of those policies was the policy on Occupational Specific Dispensation (OSD). The policy was created to cater to career pathing within the profession, and recognition of appropriate experience for grade progression (PHSDSBC 2010). However, based on the findings the policy failed to adequately recognise sub-specialities (radiation therapy, nuclear medicine, mammography and ultrasonography) within the profession but only focused on diagnostic radiography. The findings showed that there are no supervisory posts except for the assistant director’s position in sub-specialities of radiography. In addition, radiation therapists, ultra-sonographers and mammography radiographers were not recognised according to their registration with the Health Profession Council of South Africa. They were placed in diagnostic radiography posts, and this was evident in their employment package. According to managers, this was caused by the failure of the radiography profession to have a direct presentation at the Bargaining Council Chambers of South Africa.

The second policy that was associated with negative sentiments from participants was the PMDS. One of the key objectives of the policy was to improve employee performance through financial recognition linked to their PMDS (DPSA 2007). However, participants believed that the managers or supervisors did not rate them fairly, and the rating was biased towards radiographers, who were liked by their managers. Similar challenges related to rating of participants have been previously raised by other authors within nursing (Mashego & Skaal 2016). The participants believed that every radiographer should receive positive PMDS ratings because of the staff shortages resulting in heavy workloads. If radiographers receive negative PMDS ratings, they miss out on financial incentives and could remain in one salary grade for a lengthy period, resulting in demotivation and intent to seek employment in the private sector. The challenge of remaining in one salary scale for a lengthy period was also compounded by the government’s failure to pay PMDS bonuses because of financial constraints in the national fiscal.

The combination of the two policies (OSD and PMDS) was cited as a significant inhibitor to career advancement within the radiography profession by participants. If a radiographer’s PMDS ratings were rated as ‘not effective’, then they could remain in position for 10 years. Also, none of these policies acknowledges postgraduate qualification within the profession, thus demotivating postgraduate education. Participants also cited dissatisfaction with placement or rotation in different imaging or treatment machines, where they said it was biased towards those who were liked by supervisors. This resulted in losing the ability to apply the skills they would have learned at institutions of higher learning. For instance, radiotherapists cited not being placed in treatment machines. This field of radiotherapy (treatment planning) has got several novel developments such as automated planning and robust planning (Hansen et al. 2020).

### Recommendations

It is recommended that a larger study is conducted with a dedicated focus on each of the disciplines in radiography. Further studies are needed to fully explore the influence of government policies on each discipline within the radiography profession. Alternatively, a quantitative study may be conducted to determine if there is a difference in the level of satisfaction with the implementation of government policies and the lack of career pathing.

In addition to this, the current professional bodies that exist within radiography should unite or collaborate to ensure the direct representation of radiographers at the Bargaining Chamber of South Africa. Furthermore, collaborations are needed from institutions of higher learning to facilitate the recognition of postgraduate qualifications by the National Department of Health.

### Limitations

Firstly, the study was conducted in two tertiary hospitals only, and therefore the findings were limited to only these types of hospitals. Secondly, the number of radiographers who participated in the study was too small in comparison to the general population. Based on what was done in the study, the researcher took one representative from each discipline, and this could have narrowed the views. There could have been different views that would have been obtained in other hospital settings. Also, the inclusion of the different disciplines of radiography in one interview setting might have limited the discussions, whereas there might be more matters that are discipline-specific.

## Conclusion

The study was done to explore the influence of government policies on job satisfaction for radiographers employed by tertiary hospitals in the Gauteng province. Three main policies were mentioned by radiographers in the focus group interviews and in the one-on-one interviews. These were the policies on Occupational-Specific Dispensation, the PMDS and the *EEA*. The majority of the dissatisfaction with these policies was on their implementation. If the OSD and the PMDS are unfairly implemented, they would hinder career progression within the field and this could lead to salary stagnation. This would suggest that they need to be reviewed to ensure that they are consistently applied across the different disciplines of radiography.
